# Effect of an Acute Insect Preload vs. an Almond Preload on Energy Intake, Subjective Food Consumption and Intestinal Health in Healthy Young Adults

**DOI:** 10.3390/nu14071463

**Published:** 2022-03-31

**Authors:** Alba Miguéns-Gómez, Marta Sierra-Cruz, Esther Rodríguez-Gallego, Raúl Beltrán-Debón, M Teresa Blay, Ximena Terra, Montserrat Pinent, Anna Ardévol

**Affiliations:** 1 MoBioFood Research Group, Departament de Bioquímica i Biotecnologia, Universitat Rovira i Virgili, c/Marcel·lí Domingo nº1, 43007 Tarragona, Spain; alba.miguens@urv.cat (A.M.-G.); marta.sierra@urv.cat (M.S.-C.); esther.rodriguez@urv.cat (E.R.-G.); raul.beltran@urv.cat (R.B.-D.); mteresa.blay@urv.cat (M.T.B.); ximena.terra@urv.cat (X.T.); anna.ardevol@urv.cat (A.A.); 2 Institut d’Investigació Sanitària Pere Virgili (IISPV), 43007 Tarragona, Spain

**Keywords:** protein, satiety, insect, almond, appetite, food energy intake

## Abstract

Protein is considered the most satiating macronutrient, and its effect on satiety and food intake is source-dependent. For the first time, we compared the effect of the administration of an insect or almond preload, both containing 20 g of protein, on appetite and food intake in human subjects. Participants consumed both foods and a vehicle as a liquid preload on three separate days. They were then offered a breakfast and lunch buffet meal at which food intake was measured. Visual analogue scale (VAS) questionnaires were completed following the three preloads to assess appetite and other sensations. At breakfast, reduced energy intake was observed for both preloads compared with vehicle. At lunch, food intake only differed in the insect group, which consumed more than the vehicle. Insect preload increased the total amount of protein ingested with a slight increase in total energy consumed, differently than almond, which significantly increased total protein and energy consumed. There was no correlation between indigestion-sensation ratings and food intake. Moreover, the insect preload resulted in lower sleepiness and tiredness ratings compared with the almond preload. Thus, insect-derived protein may be suitable as a safe ingredient for snacks intended for elderly or infirm patients who require increased protein intake.

## 1. Introduction

Protein is regarded as the most satiating macronutrient, and high-protein diets can potentially greatly suppress hunger [1,2]. Dairy protein has been the focus of most human-study research in this area. Several studies have indicated that whey protein has a greater effect on appetite than other proteins such as egg, tuna, turkey or casein. Pal and Ellis [3] found that energy intake in an ad libitum meal was significantly lower four hours after a whey preload compared to the same dose of protein (50.8 g) from tuna, egg or turkey. Other authors have also reported that whey proteins reduce food intake more effectively than soya or egg albumin [4] and that a whey preload performs better than casein, in addition to yielding greater subjective satiety ratings [5] 60 min and 90 min after the preload (50 g of protein). However, other studies have reported an increase in food intake one hour after a preload of 20 g of whey protein compared to the same dose of pea and casein protein [6]. In comparisons of whey and soya and gluten [7] or whey and casein [8] preloads of about 50 g each, other authors have found no differences in appetite or food intake three hours later. These discordant results may be due to the fact that the source of the protein may play a role in its satiating effect, as well as the dose, time of consumption, the characteristics of the participants and the experimental design [4,6,9,10].

The most widely consumed protein today is animal protein and, consequently, it has received the most research attention. The demand for animal protein is expected to increase globally, despite the fact that its production carries numerous negative implications for the environment [11]. The search for alternative protein sources with environmentally friendly production processes, such as insects [12,13], is therefore taking on increasing importance. Some authors have described insects as a promising new food source that is rich in high-quality protein [13,14,15]. Insects also contain a substantial proportion of good-quality fats as well as numerous minerals and vitamins [16,17,18,19]. In addition, some evidence, mostly from in vitro studies or based on traditional medicinal uses, suggests that edible insects may have beneficial effects on human health related to aspects such as the interaction with microbiota [20], antihypertensive peptides [21] and antimicrobial function [22], among other benefits [23,24,25,26]. However, to our knowledge, the possible effect of insect consumption on food intake has never been studied. Until now, research has focused only on evaluating the nutritional value and the potential allergic and toxicological risks of this alternative protein source [27,28]. Previously, our group compared the acute effect of the same dose of three different proteins—insect (*Alphitobius diaperinus*), almond and beef [29]—on food intake in rats. Our results showed a higher caloric intake in the groups treated with the insect protein and a lower intake in the groups treated with the almond protein compared to the control group (water).

The purpose of this study is to test the acute effect of two of these protein sources, insects and almonds, on satiety in humans. For this purpose, we measured the food intake of healthy young participants at subsequent ad libitum breakfast and lunch meals and their subjective ratings of appetite as well as the effects of the two protein sources on intestinal transit. Our aim was also to compare these proteins in whole foods instead of as isolated proteins and to keep the texture, appearance and taste as similar as possible.

## 2. Materials and Methods

### 2.1. Subjects

Healthy men (*n* = 17) and women (*n* = 12) between the ages of 22 and 33 years with a body mass index (BMI) lower than 40 kg/m^2^ participated in the study. Volunteers were excluded if they had food allergies, had a BMI exceeding 40 kg/m^2^, did not follow the study guidelines, had a major systemic illness, used drugs to treat metabolic-related syndrome pathologies, had any intestinal related problems or were experiencing any COVID-19 symptoms. This study was approved by the ethical research committee on medicines of the Pere Virgili Health Research Institute (CEIM 172/2020, accepted on 3 August 2020). All participants provided informed, written consent before participating in the study.

### 2.2. Experimental Design

**Figure 1:**
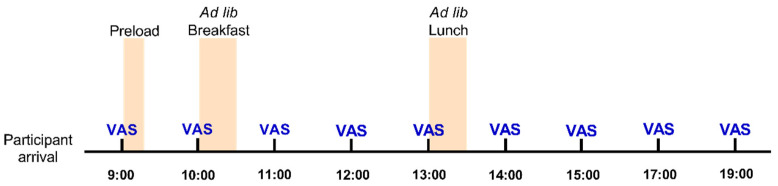
Timeline of the experimental design for each day of the study. VAS, visual analogue scale.

This was a single-blind, cross-over design study in which three different preloads were administered in a random order to all the participants in three different visits. The participants came to the study room on three separate days with a six- to seven-day period between each visit. Subjects were asked to arrive at 8:45 a.m. after a minimum fasting period of 10 h. On the first day of the study, the participants were weighed, and their heights were recorded. They were asked to follow their normal dietary habits and not to take part in any intensive physical activity on the days before study days. On each of the three occasions, the subjects received one of the preloads at 9:05 a.m. Five minutes before the preload, participants were asked to complete questionnaires, using visual analogue scales (VAS), to rate their appetite. They were asked to complete the questionnaire again every hour until the end of the study (9:00 a.m.–7:00 p.m.), as detailed in Figure 1.

The participants consumed the preload after completing the appetite questionnaires. One hour later, subjects were offered a buffet breakfast to be consumed ad libitum consisting of orange juice; chocolate-filled or plain croissants; cheese, turkey or dry sausage sandwiches; black coffee with optional milk and sugar; and bananas. Four hours after the preload (1:00 p.m.), subjects were offered a buffet lunch consisting of four varieties of pizza (barbeque, vegetarian, five-cheese or carbonara, supplied by Telepizza Group, S.A., Tarragona, Spain). We presented the buffets in identical trays containing eight portions of pizza based on the participants’ preferences. A piece of fruit, tangerine or pear, the consumption of which was mandatory, was also included. Participants were asked to eat until they felt comfortably full and were given 30 min to consume the meals. All the food was weighed before each subject started eating the buffet meals and then again afterward. The energy (kJ) content of the food consumed was determined using the information provided by the manufacturers and the Spanish food composition database BEDCA (Base de Datos Española de Composición de Alimentos) for the fruit. The volunteers were allowed to continue with their daily routine and leave the building with a commitment to return on time for the scheduled meals. Throughout the duration of the experiment, the participants were allowed to drink as much water as they wanted.

### 2.3. Preloads

On each of the three days of the study, the subjects received one of the preloads containing either insect (buffalo larvae, *Alphitobius diaperinus* powder; Protifarm NV, Ermelo, the Netherlands), almond (*Prunus dulcis* flour; Borges Agricultural & Industrial Nuts (BAIN), Reus, Spain) or no protein source (a cocoa milkshake used as a vehicle). The three preloads were prepared using a blender to mix 100% cocoa powder (Chocolates Valor, S.A., Alicante, Spain), skimmed milk, sucralose, a food colourant (brilliant blue, E133) and almond or insect flour (just for the two protein-containing preloads) to have a final content of 20 g of protein in both preloads. The nutritional composition of the preloads is described in Table 1. The test meals were matched as closely as possible in terms of appearance, texture and taste, and were tested for palatability by the laboratory staff. Subjects consumed test meals as a blended drink, flavoured with chocolate powder, to blind the subjects to the source of the protein. They were required to finish the milkshake within 5 min. They were also asked to report the time at which they first detected the colourant in their faeces in order to monitor the excretion rate of the preloads.

### 2.4. Subjective Ratings of Appetite

Appetite and other sensations were assessed using a method based on VAS [30,31]. Participants were instructed to move the cursor along a horizontal line using the mouse to indicate how they felt about each individual variable with “not at all” on the left and “very/a lot” on the right [32]. Questions about “motivation to eat” (desire to eat, hunger, prospective food consumption, fullness) alternated with questions about other sensations (thirst, stress, sleepiness, tiredness, indigestion and tummy rumbling). The complete list of questions is available in Supplementary Table S1.

To assess the palatability of the three preloads, the subjects were asked to rate, using something similar to the VAS questionnaires, the overall likeability, pleasantness of taste and likeability of texture after finishing the study.

### 2.5. Statistical Analysis

The effect of the preloads, BMI (divided into normoweight, overweight and obese), gender and their interaction on food intake measurements were tested by univariate repeated-measures analysis of variance (ANOVA) followed by the Bonferroni adjustment for multiple comparisons. To test the effect of preload, time, BMI, gender and their interaction with VAS questionnaires, we used a univariate repeated-measures ANOVA, followed by the Bonferroni adjustment for multiple comparisons to investigate differences between preloads. Gender and BMI were introduced as between-subject factors in both analyses. The area under the curve (AUC) was calculated using the trapezoidal rule. Significance was set at *p* < 0.05, unless otherwise indicated. Data are presented as means ± standard error of the means (SEMs.) All the calculations were performed using XLSTAT 2021.2.1 software (Addinsoft, New York, NY, USA).

VAS questionnaires missing values because of delayed or unreported answers were estimated using a Markov Chain Monte Carlo (MCMC) multiple imputation algorithm.

## 3. Results

### 3.1. Energy and Protein Intakes

**Figure 2:**
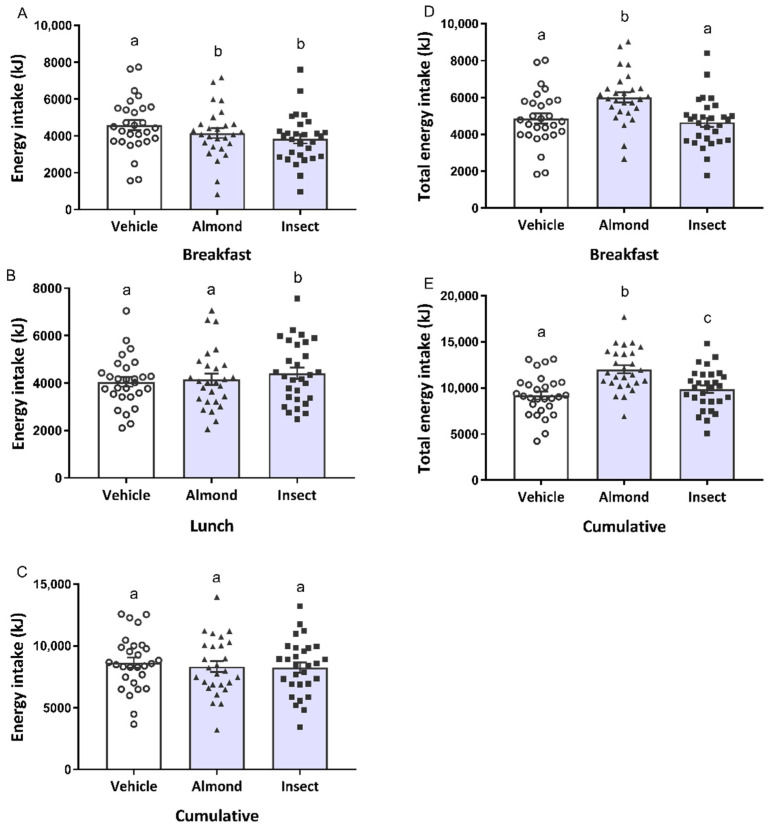
Ad libitum energy intake at the offered breakfast (**A**), lunch (**B**) and cumulative intake (**C**), which represents the energy intake from both meals together. Total energy intake, calculated as the sum of the energy from the preload and the ad libitum meal, at breakfast (**D**) and cumulative total energy intake (**E**). Participants received a dose of 20 g of protein from almond and insect preloads, together with the rest of the macronutrients from each aliment, and 5.7 g of protein from the vehicle preload, on three different occasions. Subsequent energy intake was measured at breakfast and lunch. Data are expressed as participants’ individual intake (○, vehicle; ▲, almond; ■, insect) and mean ± standard error of the mean (SEM) of the participants from each group (bars). Different letters (a,b,c) indicate significant differences, *p* < 0.05 (repeated-measures ANOVA followed by Bonferroni adjustment for multiple comparisons).

We evaluated the effect of the quality of a 20 g protein-containing preload on food intake and analysed the effect of protein source, BMI, subject gender and their interaction with the different outcomes. Ad libitum breakfast and lunch test meal intake (without including the energy of the preload) was affected by subject gender (*p* < 0.0001) and preload (*p* = 0.007 and *p* = 0.02, respectively). Since there was no interaction between preload and subject gender or preload and BMI (*p* > 0.05 in all the cases), the results for the different BMI groups and both genders are presented together. Breakfast test meal intake from both the almond- and insect-treated groups was significantly lower than the vehicle-treated group (Figure 2A). At lunch, four hours after preload administration, we observed a different profile: The insect-administered group ate more than the almond or vehicle groups (Figure 2B). Cumulative energy intake, calculated as the sum of energy from breakfast and lunch, was not significantly different among the treatments (Figure 2C).

We observed a gender (*p* < 0.001) and preload interaction (*p* < 0.0001) with total energy intake, calculated as the sum of energy from the preload and the ad libitum meal, for the breakfast test meal and cumulative intake. However, since no interaction was found between preload and subject gender or between preload and BMI (*p* > 0.5), we did not separate the results for both genders and for the three BMI categories. Total energy intake for the almond-administered group was significantly higher than that of both the vehicle and insect groups at breakfast (Figure 2D). A preload effect was observed in the total cumulative energy intake after lunch. The almond-administered group exhibited the highest energy intake, followed by the insect and vehicle groups (Figure 2E). No differences were observed between the vehicle and insect groups, but the difference between the insect and almond groups was significant.

**Figure 3:**
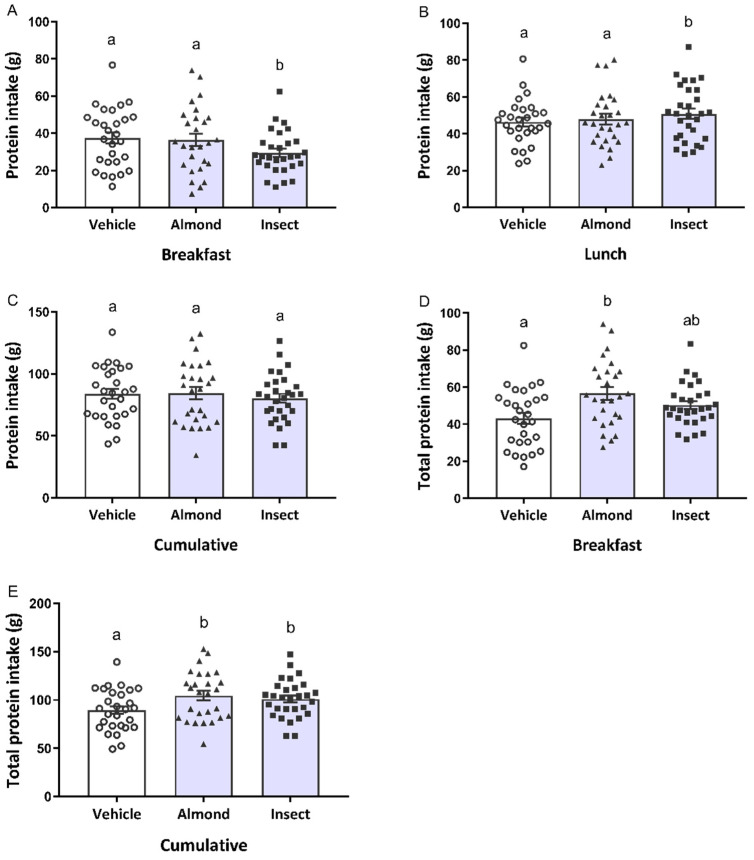
Ad libitum protein intake at breakfast (**A**), lunch (**B**) and cumulative protein intake (**C**), calculated as the sum of the protein grams eaten at breakfast and at lunch. Total protein intake, calculated as the sum of the protein from the preload and the ad libitum meal, at breakfast (**D**) and the total cumulative protein intake (**E**). Data are expressed as participants’ individual intake (○, vehicle; ▲, almond; ■, insect) and mean ± standard error of the mean (SEM) of the participants from each group (bars). Different letters (a,b) indicate significant differences, *p* < 0.05 (repeated-measures ANOVA followed by Bonferroni adjustment for multiple comparisons).

Since we administered the same amount of protein in both protein-containing preloads, we compared their effect on protein intake. Again, we found no interaction between preload and subject gender and preload and BMI (*p* > 0.05). For the ad libitum protein intake, a different profile was observed compared to the energy intake at breakfast. We detected a preload effect (*p* = 0.012) in that the insect-administered group ate less protein than the almond or vehicle groups (Figure 3A). In this case, the almond-administered group consumed the same amount of protein as the vehicle group, despite having lower energy consumption. At lunch, we observed the same profile as for energy intake: A higher protein intake only in the insect-administered group (Figure 3B). No preload effect was found in the cumulated protein intake (Figure 3C), calculated as the sum of protein grams eaten at breakfast and at lunch.

We also observed a preload effect at breakfast (*p* < 0.0001) with regard to total protein intake, calculated by adding the protein present in the preloads (20 and 5 g of protein for the protein-administered groups and the vehicle group, respectively) to the ad libitum protein intake. Here, the almond group had a higher protein intake than the vehicle group (Figure 3D). A preload effect was also observed (*p* < 0.0001) for the accumulated total protein intake since both the insect- and almond-administered groups presented a higher total protein intake compared to the vehicle group. No differences were found between the two protein-administered groups (Figure 3E).

### 3.2. Subjective Ratings of Appetite

Complementary to energy and protein intake measurements, subjective ratings of appetite (measured with VAS questionnaires) were collected over the course of each test day at the times indicated in the section on our experimental design (Figure 1). As in other analyses, none of the results obtained showed an interaction between preload and subject gender or between preload and BMI, so the data presented here are combined for both genders and the three BMI categories.

**Figure 4:**
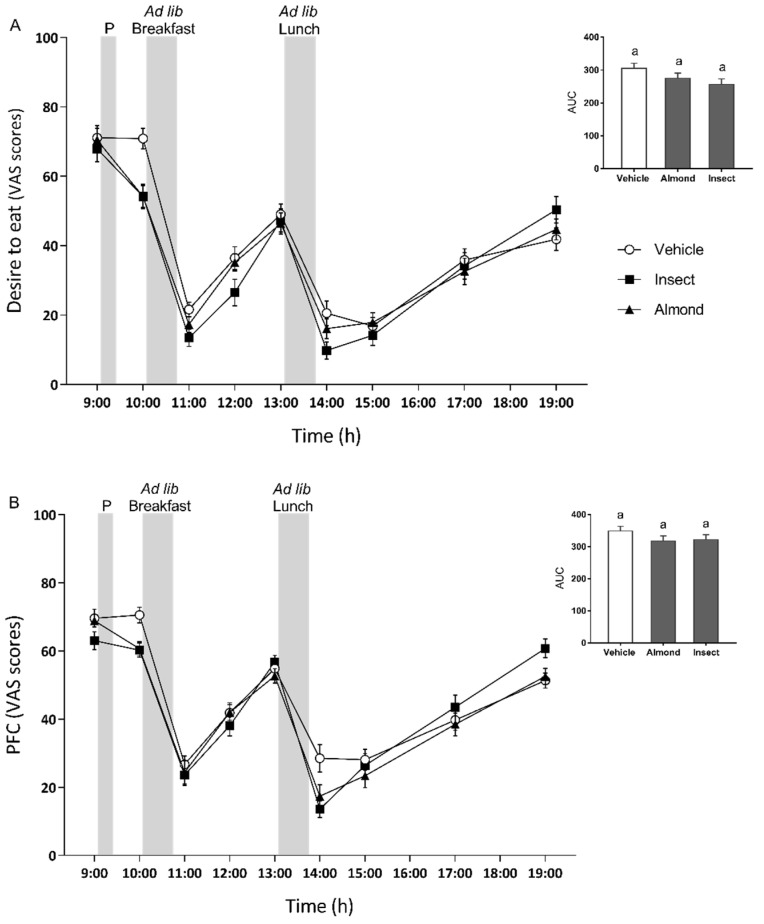
Desire to eat (**A**) and prospective food consumption (**B**) ratings during the three test days. Grey bars indicate the times of the preload (P), breakfast and lunch intake. Participants were asked to complete VAS questionnaires to rate their motivation to eat five minutes before P and at the subsequent time points indicated in the graph. Data are expressed as mean ± standard error of the mean (SEM). The bar graph represents the mean AUC of each of the three preload groups for both variables. Same letters (a) indicate no significant differences, *p* > 0.05 (repeated-measures ANOVA followed by Bonferroni adjustment for multiple comparisons).

As expected, one hour after the ad libitum test meals (both breakfast and lunch), the desire to eat and prospective food consumption ratings (Figure 4) were lower for all three groups compared with the time points just before the meals were given, that is three and six hours after preload administration (time effect, *p* < 0.001).

The temporal profile for the desire to eat is shown in Figure 4A. The type of protein ingested had an effect on this factor (*p* = 0.035): The desire to eat was lower after the insect preload than after vehicle consumption. A strong interaction between time and preload by time (*p* < 0.006) was also observed. The desire to eat was significantly lower one hour after preload administration (just before breakfast) for both the insect and almond groups. These ratings predicted what we observed during breakfast intake, where both protein-administered groups consumed fewer calories than the vehicle group. Only the insect-administered group reported lower ratings regarding the desire to eat one hour after breakfast (two hours after preload administration) than the vehicle group.

Ratings for prospective food consumption (Figure 4B) were independent of the type of preload administered (*p* = 0.076), but they were affected by time and the preload-by-time interaction (*p* < 0.0001 and *p* = 0.001, respectively). Both the insect- and almond-administered groups reported that they could eat less one and five hours after the preload than the vehicle group did. These differences were statistically significant. At the end of the study, ten hours after the administration of the preload, the insect-administered group thought they could eat more than the almond- or vehicle-administered groups.

The participants were asked to answer additional questions about their motivation to eat as well as other sensations in the VAS questionnaires. We analysed stress, tiredness, sleepiness, thirst, and tummy rumbling. Only the tummy-rumbling question showed a preload-by-subject-gender interaction. Therefore, all the data for both genders are presented together except for this question. These results are shown in Supplementary Figures S1–S5 of the Supplementary Material. The hunger and fullness questions were excluded from the analysis as they were not statistically valid.

A preload effect was observed (*p* = 0.042) for the stress ratings: The insect group felt less stressed than the vehicle group. One hour after the breakfast and lunch test meals, both the insect- and almond-administered groups felt significantly less stressed than the vehicle group. A preload interaction was observed (*p* < 0.0001) for the feeling of tiredness, with lower tiredness ratings reported for the insect-administered group than for the almond- or vehicle-administered groups. One hour after breakfast and one hour after lunch, both the vehicle- and almond-administered groups felt significantly more tired than the insect group. Moreover, the almond group felt sleepier one hour after the breakfast and lunch test meals than the insect group. For the sleepiness ratings, we observed a preload effect (*p* = 0.003) in which the insect group felt less sleepy than the almond group. We also observed a preload interaction (*p* = 0.012) in participants’ thirst ratings after the preload intake. The insect-administered group reported greater thirst than the vehicle group. Finally, we observed a preload interaction (*p* < 0.001) in the men’s tummy-rumbling scores that showed that the insect group had lower ratings than the vehicle or almond groups. No preload effect was observed for the women’s scores.

Although specific time point differences between the three preloads were observed for some questions (detailed in the Supplementary Material, Supplementary Figures S1–S5), no preload-by-time interaction was observed for any of the answered questions.

### 3.3. Preload Palatability and Correlation with Food Intake

The participants were offered a cocoa milkshake containing almond, insect or no protein source (vehicle). The flavour and texture of the three drinks were as similar as possible. Nonetheless, we wanted to check the potential effect of the palatability of the preload on subsequent food intake. To this end, the participants were asked to rate the overall likeability, pleasantness of taste and the likeability of the texture by completing VAS type questionnaires scaled from 0 to 10. We found that the preload affected the ratings of overall likeability, taste and texture of the preloads (*p* = 0.001, *p* = 0.006 and *p* < 0.0001, respectively). Nevertheless, there were no significant differences between the two protein-containing preloads regarding these three parameters (Table 2). The vehicle preload obtained significantly higher ratings for texture and overall likeability compared with the two protein-containing preloads and slightly higher ratings for the pleasantness of taste compared to the insect preload.

The relationship between the palatability of the preloads and subsequent energy intake was tested using Pearson’s correlation analysis. Food energy intake during the test meals, at breakfast and lunch, was not correlated with the measures of the pleasantness of taste (*r* = 0.152 and *r* = 0.104, respectively), overall likeability (*r* = 0.158 and *r* = −0.002, respectively) or likeability of texture (*r* = 0.196 and *r* = −0.061, respectively) for any of the three preloads (*p* > 0.05 in all cases).

### 3.4. Digestive Health and Its Correlation with Food Intake

**Figure 5:**
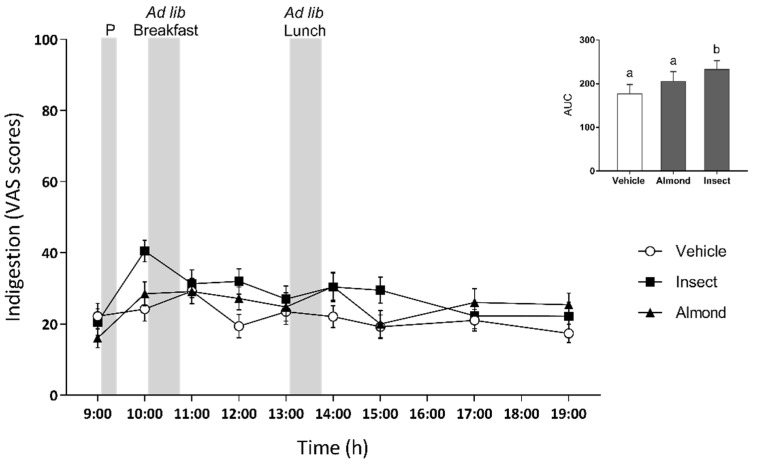
Indigestion ratings during the three test days. Grey bars indicate the time of the preload (P), breakfast, and lunch intake. Data are expressed as mean ± standard error of the mean (SEM). The bar graph represents the mean AUC of each of the three preload groups for the indigestion ratings. Different letters (a,b) indicate significant differences, *p* < 0.05, (repeated-measures ANOVA followed by Bonferroni adjustment for multiple comparisons).

As we tested a non-conventional food product, we wanted to monitor possible side effects at the gastrointestinal level. First, we worked with the VAS questionnaire in which participants were asked to indicate any feeling of indigestion (Figure 5). The questionnaire monitored any side effects following the consumption of the preload and test meals at each time point when the appetite VAS questionnaires were completed. After the consumption of each preload, we observed a significant effect of time (*p* < 0.001) and the preload-by-time interaction (*p* = 0.038) throughout the study period. We also observed a preload effect (*p* = 0.007) in which the insect preload gave rise to a greater sensation of indigestion than the vehicle. Nevertheless, Pearson’s correlation analysis indicated that there was no relationship between the intake at breakfast and the indigestion scores just before that meal (*r* = −0.034, *p* = 0.756). However, we observed a positive correlation between the intake at lunch and the indigestion scores just before that meal (*r* = 0.236, *p* = 0.031).

**Figure 6:**
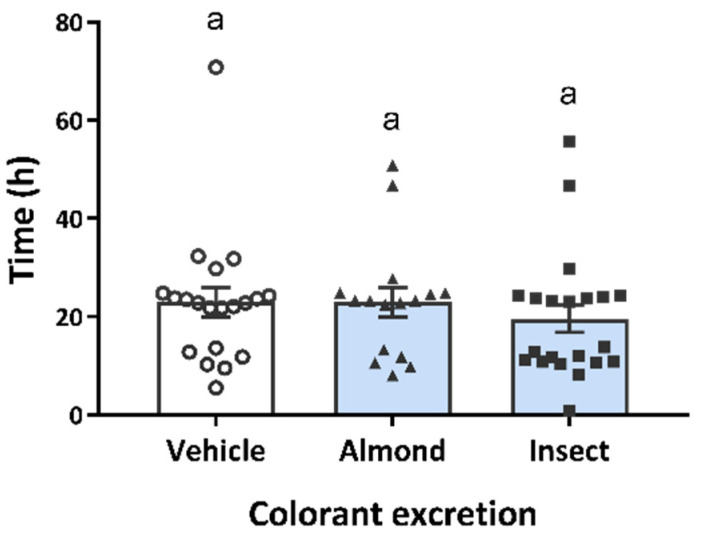
Colourant excretion in faeces. Participants were asked to report the time at which they noticed colourant in their stool. Data are expressed as participants’ individual intake (○, vehicle; ▲, almond; ■, insect) and mean ± SEM of the participants from each group (bars). Same letters (a) indicate no significant differences, *p* > 0.05 (Repeated-measures ANOVA followed by Bonferroni adjustment for multiple comparisons).

Secondly, since we are comparing two protein sources with different fibre matrices and protein qualities (Accardo, F. et al., 2022, submitted), we also measured how the preload might affect intestinal tract mobility. As with other variables, there was no interaction between gender and the preload (*p* = 0.347), so data for both genders are presented together. In this case, we observed a subject gender effect (*p* = 0.026). Figure 6 shows that there was no preload effect in relation to gender (*p* = 0.936). What we found was a strong negative correlation (*r* = −0.345, *p* = 0.009) between intestinal mobility after the preload intake and the indigestion AUC, suggesting that a greater sensation of indigestion induces shorter intestinal transit.

## 4. Discussion

This is the first study to evaluate the effect of insect consumption on food intake in humans. As expected, both almond and insect preloads (20 g) reduced food intake more than the vehicle (non-protein preload, 5.7 g) one hour after consumption at breakfast (Figure 2A). Nevertheless, no differences were found between the two protein-containing preloads even though the almond preload contained 2.3 times more energy than the insect preload. The same occurred at lunch when the almond group ate the same amount as the vehicle-administered group despite having 6.6 times more energy content (Figure 2B). This might be explained by the low energy absorption [33] and low protein bioavailability [34] described for almonds. Meanwhile, the preload containing insect exhibited a different profile at lunch, increasing food intake at that time point compared to the vehicle and almond groups. This increased food intake was previously observed in rats 20 h after the oral administration of an insect protein dose [29], and in humans when comparing a high energy-dense preload with a low energy-dense preload of the same weight [35]. It is thought that the chitin present in insects affects protein digestibility [36] and may interfere with the absorption of other nutrients. However, in a previous analysis by our group in which we worked with the same samples used in this study, we found that the digestibility and bioaccessibility of insect protein are better than those of almonds (Accardo, F. et al., 2022, submitted). Another explanation might be compensation for the lower energy intake at breakfast. In fact, when we quantified the cumulative energy intake (breakfast and lunch together), the differences in food intake during the two meals disappeared. This indicates that the participants compensated for the energy consumed within the preload, for both preloads, similarly to that reported in Hull, S. et al. [37] after a mid-morning almond snack.

Several studies have shown that palatability affects satiation, that is the amount eaten within a meal, with an increased intake as palatability increases [38,39]. As a certain food is eaten, the palatability starts declining and it becomes less likely that it will be eaten, a phenomenon called sensory-specific satiation [40]. Although its duration is not specified, different studies have shown that the reduced liking of the food can be maintained until 1 h after consumption [41]. Nevertheless, this reduction in the pleasantness of food disappears if eating a different aliment. In our study, we wanted to check that the palatability of the preloads was not affecting satiety, that is, subsequent food intake. The palatability of both preloads obtained lower ratings than the vehicle. However, these ratings were not correlated with food intake at breakfast or at lunch, suggesting that the palatability of the preloads did not influence subsequent food intake as previously described by other authors [42,43,44]. We also wanted to rule out the possibility that food intake was affected by any side effects caused by the preload. However, as we have shown, although the insect preload obtained higher ratings for the sensation of indigestion (at 10:00 a.m.) than the vehicle did, these were not correlated with food intake at breakfast (*r* = −0.034, *p* = 0.756). On the other hand, a positive correlation was observed between indigestion scores at 1:00 p.m. and food intake at lunch, where we observed increased food intake (*r* = 0.236, *p* = 0.031). A strong negative correlation was also observed between the colourant excretion rate and the indigestion ratings. Nevertheless, no differences between the three preloads were found regarding this aspect (Figure 6), and together with the absence of correlation between indigestion and food intake, we can say that the preloads did not cause any side effects, as reported by other authors [20] for cricket consumption.

The mechanisms underlying the satiety process have not yet been fully described. Different pathways are involved, and how they work together is still under debate. For that reason, discrepancies in food intake after an equivalent protein dose administration are not new in satiety studies. As an example, whey has been reported to reduce food intake compared with tuna, egg and, turkey [3], when compared with soya and gluten [4] and with casein [5]. However, increased food intake after a whey preload has been also reported compared with pea and casein [6]. Taken together, this evidence, along with the scarce information about insects and their effect after consumption, reinforces the hypothesis that the effects on satiation of a protein dose do not depend only on the quantity, but also on the quality, dose, administration time, bioavailability and the experimental design.

In this study, we decided to include a VAS questionnaire, a reproducible and established method to gather data on appetite-related sensations, thus allowing us to conduct a more complete analysis. “Desire to eat” and “prospective food consumption” ratings at 10:00 a.m. were in accordance with the reduced energy intake at breakfast after both protein-containing preloads compared with the vehicle (Figure 4). VAS ratings of appetite and their ability to predict subsequent food intake in young subjects have been demonstrated in several studies [30,31]. Nevertheless, in our study, this approach failed to predict the subsequent food intake at lunch. The non-motivation-to-eat questions were given lower ratings by the insect group for stress, compared with the vehicle group; tiredness, compared with the almond and vehicle groups; sleepiness, compared with the almond group; and tummy rumbling (only in men), compared with both the almond and the vehicle groups. These results indicate that the almond preload seemed to make the participants feel more tired and sleepier than the insect preload, probably because of the greater amount of energy consumed, as reported by Wells, A.S. et al. for subjects two to three hours after a high-fat meal [45].

Other authors have found that the consumption of extra protein reduced the appeal of subsequent high-protein foods, causing an aversion or a regulation of protein intake [46,47]. Griffioen-Rosse et al. [48] reported this effect in a subsequent meal when comparing the protein content between their high- and low-protein meals of 13 g, similar to the 15 g difference between our protein-containing preloads and the vehicle, 30 min after preload administration. We reproduced this effect at breakfast, but only with the insect preload. After the insect preload, participants had lower protein intakes than participants in either the almond or vehicle groups (Figure 3A). As we were administering the same amount of protein to the insect and almond groups, we did not expect to find differences between them in the amount of protein consumed at breakfast. This effect was compensated for at lunchtime, resulting in no differences in the cumulative amount of ingested protein for either preload.

Taken together, these results suggest that these two preloads present different profiles. An almond preload increases both total protein and energy intake after an acute administration. Nevertheless, many studies support the endorsement of almonds as a suitable healthy snack for weight management, as no body weight or food intake increase have been reported after chronic almond consumption [49,50]. In addition to the high protein content of almonds, they also contain a substantial amount of healthy fatty acids and fibre [51]. Meanwhile, the edible insect *A. diaperinus* could be used as a product to increase protein intake, since we have described its ability to increase total protein intake with a slight increase in the total energy intake (Figure 2E and Figure 3E). Insects are a new food, and little information has been published about their effect when consumed. It has high nutritional value [14,15,16,17,18,19], and no adverse side effects were reported after the consumption of an insect preload in this study, which is supported by its extensive traditional consumption in other areas of the world, such as Latin America, Asia and Africa [52,53]. Since it has been described as being as healthy as meat products [54], and given the results obtained in this study, it may serve as a promising ingredient for snacks suitable for elderly or infirm patients who would benefit from increased protein intake.

In conclusion, our results showed that almond and *A. diaperinus* preloads modify food intake differently. Both were useful in increasing total protein intake, but the insect source was able to maintain that intake with only a slight increase in energy intake.

## Figures and Tables

**Table 1:** Nutritional composition and content of the three preloads.

Preload	Vehicle	Almond	Insect
**Nutritional analysis**			
Amount (g)	145	205	170
Energy (kJ)	279.2	1854.2	812.2
Fat (g)	1	34	8.2
Carbohydrate (g)	7.7	10.3	8.4
Fibre (g)		8.6	0.9
Protein (g)	5.7	20.1	20.7
**Content**			
Test food (g)		60	25
Cocoa powder (g)	5	5	5
Sucralose (g)	0.025	0.025	0.025
Milk (g)	140	140	140
Food colourant (mL)	0.5	0.5	0.5

**Table 2:** Palatability scores (0–10) for the three preloads containing either insect, almond or no protein source (vehicle).

Preload Type	Vehicle	Insect	Almond
Overall likeability of preload meal	7.98 ± 0.24 ^a^	5.34 ± 0.28 ^b^	5.36 ± 0.28 ^b^
Pleasantness of taste	7.61 ± 0.40 ^a^	5. 34 ± 0.49 ^b^	6.14 ± 0.39 ^a,b^
Likeability of texture	8.46 ± 0.38 ^a^	4.00 ± 0.45 ^b^	3.21 ± 0.45 ^b^

All values are means ± standard error of the means (SEMs). Means in a row with different superscript letters (^a,b^) were significantly different (Repeated-measures ANOVA followed by Bonferroni adjustment for multiple comparisons, *p* < 0.01).

## Data Availability

Not applicable.

## Supplementary Materials

The following supporting information can be downloaded at: https://www.mdpi.com/article/10.3390/nu14071463/s1, Figure S1: Stress ratings during the three test days; Figure S2: Tiredness ratings during the three test days; Figure S3: Thirst ratings during the three test days; Figure S4: Sleepiness ratings during the three test days; Figure S5: Tummy rumbling ratings during the three test days in men (**A**) and women (**B**); Table S1: Questions included in the VAS questionnaire.
