# Supplementary material for: Effect of an Acute Insect Preload vs. an Almond Preload on Energy Intake, Subjective Food Consumption and Intestinal Health in Healthy Young Adults
Source: Nutrients. 2022 Mar 31;14(7):1463. doi: 10.3390/nu14071463 (PMC9002463; doi:10.3390/nu14071463)
Supplement: Supplementary file 1 [file nutrients-14-01463-s001.zip › nutrients-1649306-supplementary.pdf]

Supplementary Table S1. Questions included in the VAS questionnaire.

Motivation to Eat	Other Sensations
How strong is your desire to eat?	I feel anxious
How hungry do you feel?	I am tired
How much do you think you could eat?	I have indigestion
How full do you feel?	I feel sleepy
	How thirsty do you feel?
	My tummy is rumbling

VAS, Visual Analogue Scale.

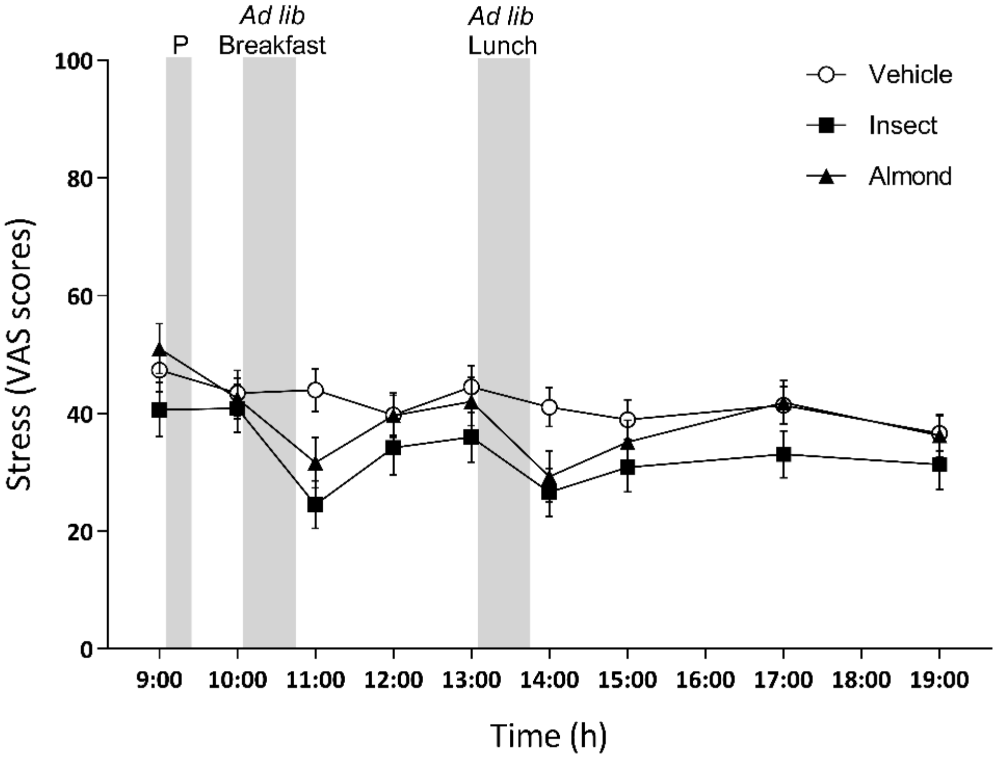

Supplementary Figure S1. Stress ratings during the three test days. Grey bars indicate the times of the preload (P), breakfast, and lunch intake. There was a preload interaction ($p = 0.042$), where the insect group felt less stressed compared with the vehicle group. No differences were observed between almond and the other groups. One hour after breakfast and one hour after lunch, both insect and almond administered groups significantly felt less stressed compared with the vehicle group. Nevertheless, there was no preload by time interaction ($p = 0.122$) but there was a strong time interaction ($p < 0.0001$). Data are expressed as mean ± standard error of the mean (SEM). Repeated-measures ANOVA followed by Bonferroni adjustment for multiple comparisons were used.

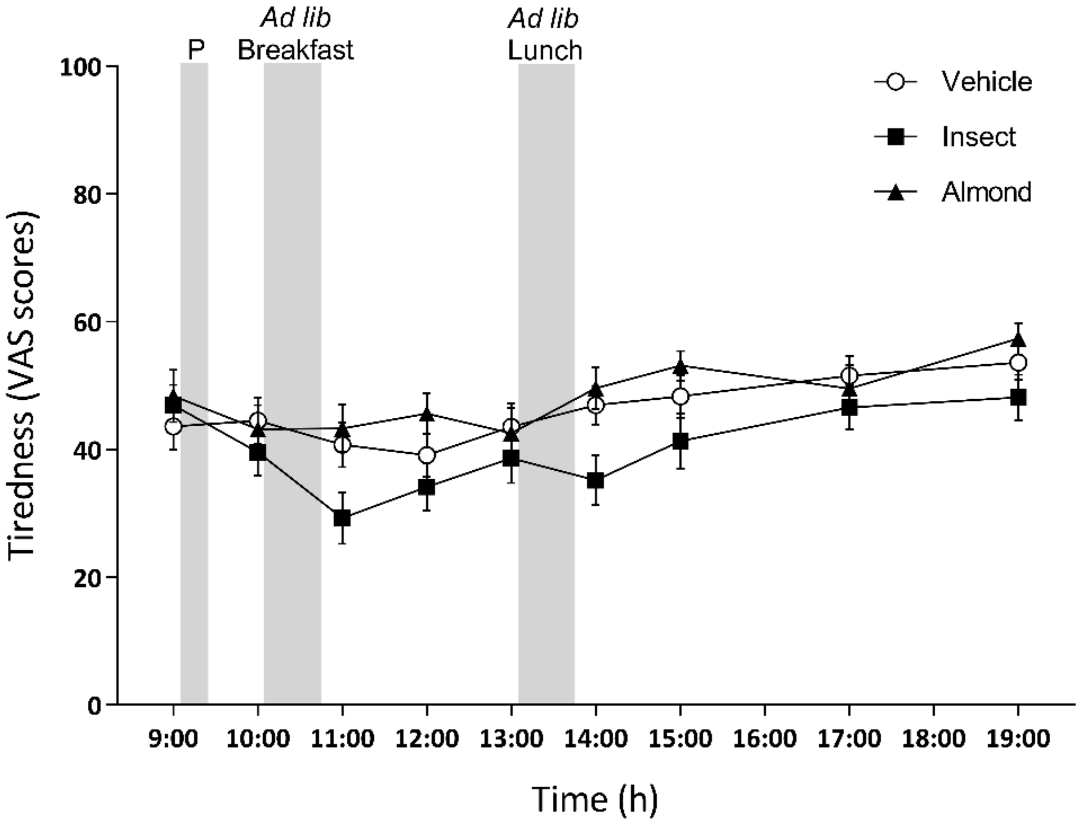

Supplementary Figure S2. Tiredness ratings during the three test days. Grey bars indicate the times of the preload (P), breakfast, and lunch intake. There was a preload interaction ($p < 0.0001$) with higher tiredness ratings for almond and vehicle administered groups compared with insect administered group. No differences were observed between vehicle and almond groups. One hour after breakfast and one hour after lunch, both vehicle and almond administered groups significantly felt more tired compared with the insect group. Three and six hours after the preload administration, just almond administered group felt more tired compared with the insect group. Nevertheless, there was no preload by time interaction ($p = 0.184$) but there was a strong time interaction ($p < 0.0001$). Data are expressed as mean ± standard error of the mean (SEM). Repeated-measures ANOVA followed by Bonferroni adjustment for multiple comparisons were used.

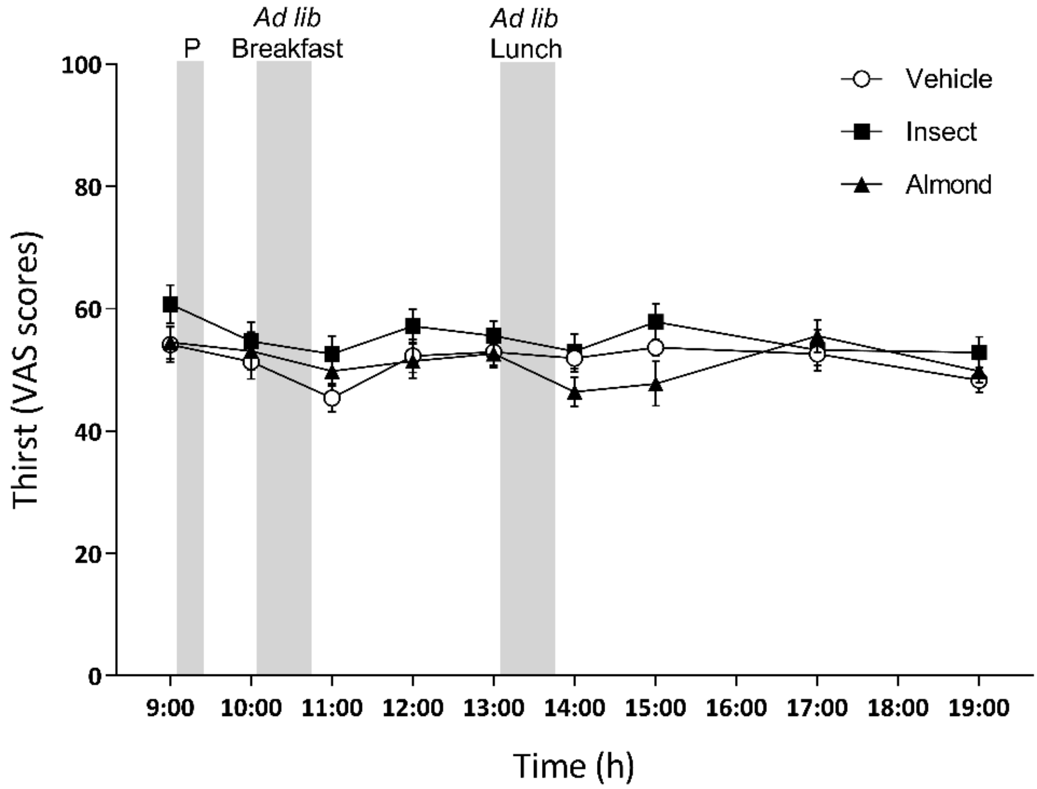

Supplementary Figure S3. Thirst ratings during the three test days. Grey bars indicate the times of the preload (P), breakfast, and lunch intake. There was a preload interaction ($p = 0.012$) with the insect administered group being the one with higher ratings compared with the vehicle group. A time interaction ($p < 0.0001$), but no a preload by time interaction ($p = 0.232$), was also observed. Data are expressed as mean ± standard error of the mean (SEM). Repeated-measures ANOVA followed by Bonferroni adjustment for multiple comparisons were used.

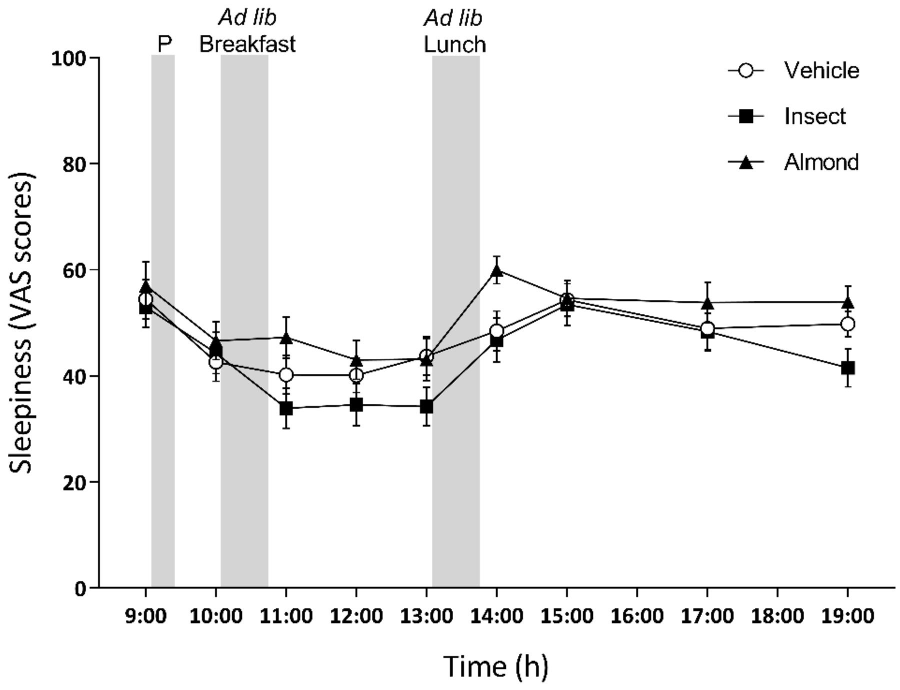

Supplementary Figure S4. Sleepiness ratings during the three test days. Grey bars indicate the times of the preload (P), breakfast, and lunch intake. There was a preload interaction ($p = 0.003$) whereby insect administered group felt less sleepy than the almond group. One hour after the test meals (both breakfast and lunch) and at the end of the study (10 hours after the preload administration) the insect administered group significantly felt less sleepy compared with almond group.

The insect group was also feeling less sleepy compared with the vehicle group just before lunch. Nevertheless, there was no preload by time interaction ($p = 0.238$). In contrast, there was a strong time interaction ($p < 0.0001$). Data are expressed as mean ± standard error of the mean (SEM). Repeated-measures ANOVA followed by Bonferroni adjustment for multiple comparisons were used.

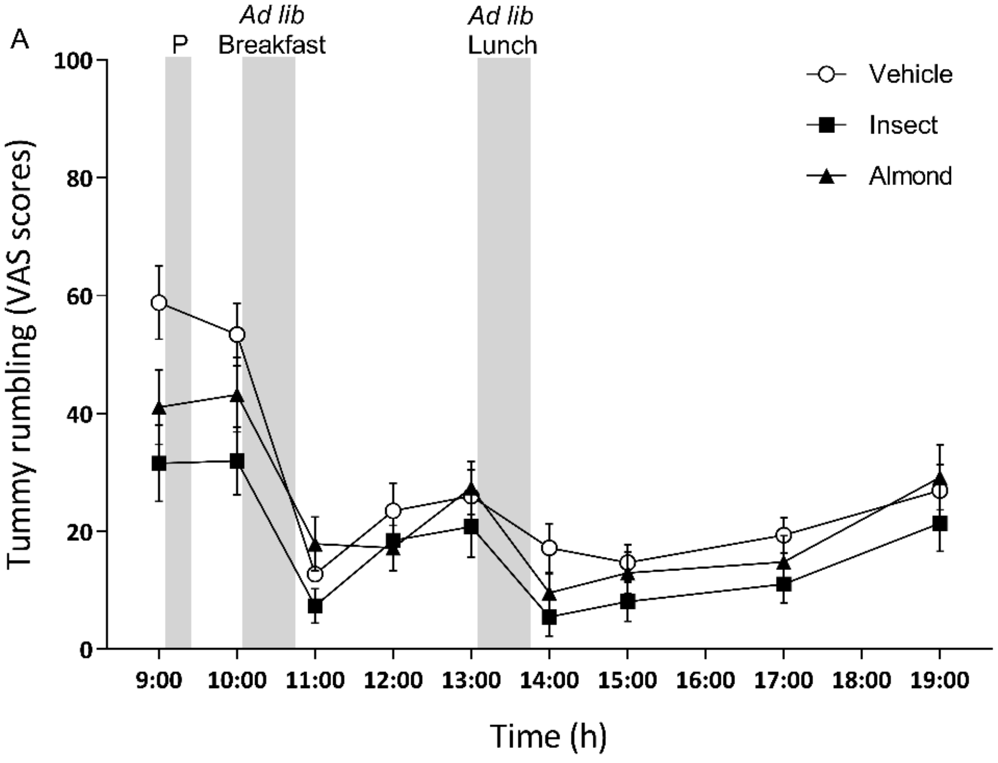

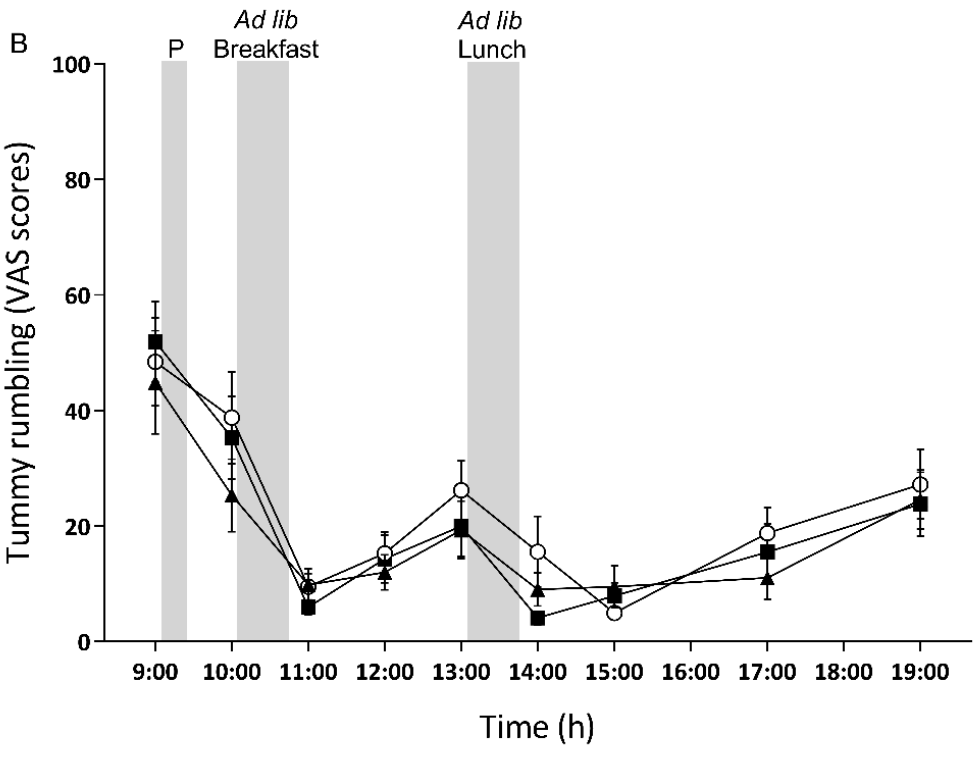

Supplementary Figure S5. Tummy rumbling ratings during the three test days in men (**A**) and women (**B**). Grey bars indicate the times of the preload (P), breakfast, and lunch intake. There was no gender effect ($p = 0.426$) but there was a preload by gender interaction ($p = 0.025$). Thus, we present these results separated for both genders. In men we observed a preload interaction ($p < 0.001$) that showed that the insect administered group had the lower ratings compared with

vehicle and almond groups. We also observed a time interaction ($p < 0.0001$) but no preload by time interaction ($p = 0.123$). In women we also observed a time interaction ($p < 0.0001$), as in the men group, but no preload or preload by time interaction was observed ($p = 0.204$ and $p = 0.581$ respectively). Data are expressed as mean ± standard error of the mean (SEM). Repeated-measures ANOVA followed by Bonferroni adjustment for multiple comparisons were used.
